# Prophylactic laparoscopic omentopexy: a new technique for peritoneal dialysis catheter placement

**DOI:** 10.1080/0886022X.2019.1583580

**Published:** 2019-03-26

**Authors:** Wei Cao, Chuanquan Tu, Tao Jia, Congjiang Liu, Liyuan Zhang, Baiqiao Zhao, Jianhua Liu, Lei Zhang

**Affiliations:** a Department of Nephrology, The First Affiliated Hospital of KangDa College of Nanjing Medical University, The First People's Hospital of Lianyungang, Lianyungang, China;; b Department of Urology, The First Affiliated Hospital of KangDa College of Nanjing Medical University, The First People's Hospital of Lianyungang, Lianyungang, China;; c Department of Haematology, The First Affiliated Hospital of KangDa College of Nanjing Medical University, The First People's Hospital of Lianyungang, Lianyungang, China

**Keywords:** Peritoneal dialysis, omentopexy, laparoscopy

## Abstract

**Background:** Prophylactic laparoscopic omentopexy is a safe technique to prevent catheter obstruction during peritoneal dialysis (PD). Here we described a technique through which the omentopexy was accomplished by Hem-o-loks before PD catheter insertion.

**Methods:** The procedures of omentopexy were described. To evaluate the efficiency of this surgical method, a retrospective review of PD catheter insertion cases and their follow-ups were performed, covering 10 consecutive patients with end-stage renal disease.

**Results:** All patients showed no intraoperative events. No catheter flow obstruction, migration, exit-site infection appeared during the follow-up.

**Conclusion:** Laparoscopic PD catheter insertion using omentopexy can decrease catheter obstruction and migration.

## Introduction

Peritoneal dialysis (PD) provides an effective and autonomously operated renal replacement therapy for patients with end-stage renal disease (ESRD) [1–5]. However, the efficacy of PD can be guaranteed only when the PD catheter is functional and durable enough to allow dialysis solution flow in and out smoothly in a long run. Complications such as catheter obstruction, migration, pericannular leak, peritonitis, and exit-site infection can lead to catheter dysfunction, even force the patient only to survive with hemodialysis [6]. An analysis of a large incident cohort of PD-dependent patients in the US suggested that the catheter insertion techniques decided the therapeutic efficacy of PD [7]. To address these complications, catheter insertion techniques have been modified in recent years [8].

Traditionally, a PD catheter is inserted by open or percutaneous techniques leaves the operator blind to the location of the catheter. Laparoscopy is always performed with proactive techniques which are not conventionally used. It enables a clear laparoscopic visualization of the adhesions and hernias in the peritoneal cavity. Limited literature has described the laparoscopic omentopexy during the insertion of a PD catheter [9–11]. With laparoscopic omentopexy, the great omentum can be grasped and moved to the target part of the abdomen, and fixed onto the parietal peritoneum. In this paper, we described a PD catheter insertion technique – prophylactic laparoscopic omentopexy – through which the greater omentum was fixed onto the omentum below the great curvature of the stomach with several Hem-o-loks.

## Materials and methods

### Patients

A total of 10 ESRD patients were recruited from The First People’s Hospital of Lianyungang Affiliated with Nanjing Medical University from May 2017 to June 2018. All patients had no history of renal replacement therapy, received written informed consent, and were informed of the operation procedures, possible complications, and alternative operating methods. The study was approved by the ethics committee of our hospital (IRB approval number: LW20170102001). All PD catheters were inserted by one experienced surgeon using the same surgical devices.

### Preoperative preparation

All patients fasted for at least eight hours and emptied their bladder before surgery. Abdominal skin preparation was finished before surgery, without any use of prophylactic antibiotics and bowel preparation.

### Operative technique (omentum fixation and catheter insertion)

The laparoscopic procedures were performed under general anesthesia. Omentopexy and catheter fixation were accomplished using one trocar as the observing port and other two trocars as the operating ports. The two trocars were used to fold and fix omentum and the PD catheter.

There were three surgical steps. The first step was fixing omentum folds, the second was suturing of sigmoid mesentery and lateral peritoneum, and the last step was inserting PD catheter into the Douglas fossa through the left side of the sigmoid colon below the suture between sigmoid mesentery and the lateral peritoneum.

Before the insertion of the camera port, pneumoperitoneum was induced using a Veress needle inserted through a point 10 mm below the umbilicus (point A). Pressure for abdominal gas insufflation was limited within 12 mm Hg. Preliminary laparoscopy was performed to look for adhesions and/or other anatomic abnormalities that could undermine the function of the PD catheter.

**Figure 1.**
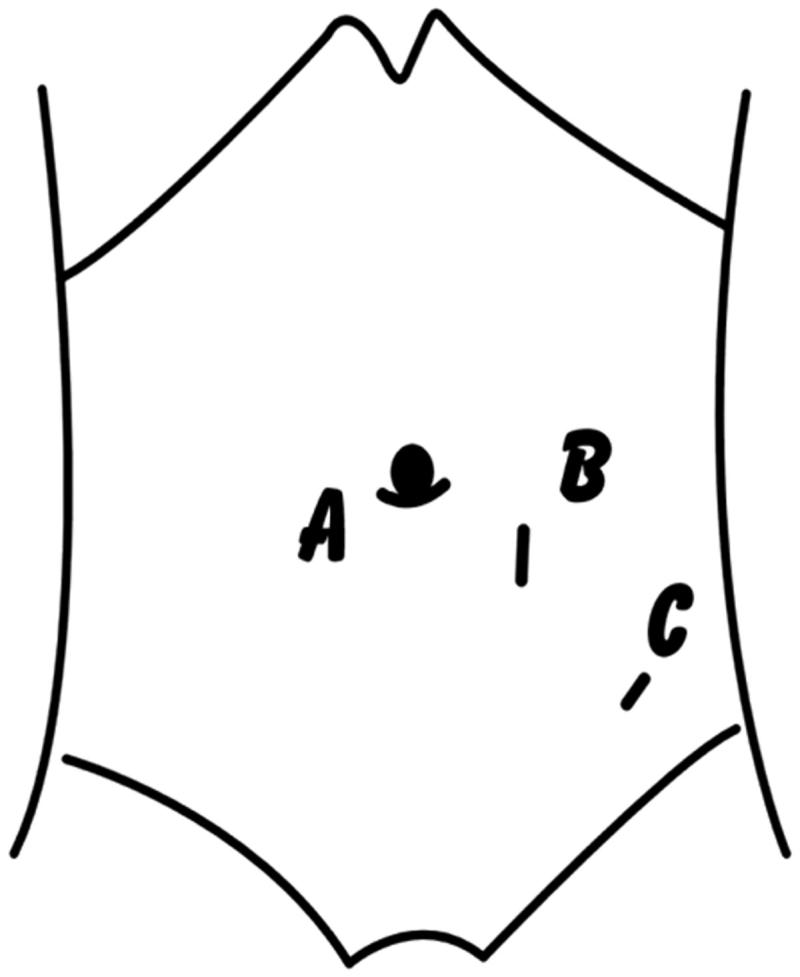
The position of three trocars at the abdominal. (A) A point 10 mm below the umbilicus; (B) 9–13 cm above the pubic symphysis and on the left paraspinal median line; (C) 6–8 cm below and left to point B.

Considering the pubic symphysis is a palpable landmark representing the upper anterior border of the pelvis and also a surface landmark for the catheter tip, the point to insert trocar B was set 9–13 cm above the pubic symphysis and on the left paraspinal median line. Point C to pass the catheter outlet was located 6–8 cm below and left to point B, and 2–3 cm to catheter’s outer cuff (Figure 1).

Under laparoscopy, a 12-mm trocar and a 5-mm trocar were inserted into the abdominal cavity through point B and C. Point B was chosen as the observation port first, point A and C as the operating ports to allow grasper and scissors.

**Figure 2.**
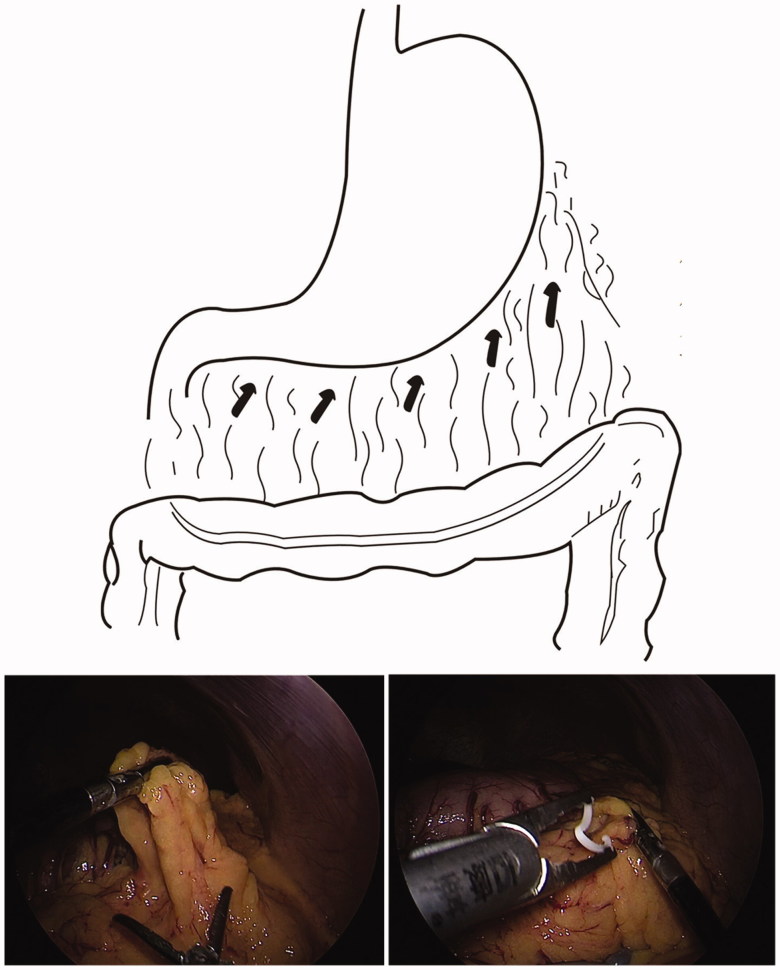
The redundant end of the omentum will be transferred into the upper abdomen by a grasping forceps that are introduced from port (A and C), then fixed it onto the omentum below the great curvature of the stomach by Hem-o-loks. (The top is a diagram and the bottom are laparoscopic views).

The omentum was checked first. The midriff and hypogastric omentum were pulled and folded to the epigastrium, and then fixed onto the omentum below the great curvature of the stomach with Hem-o-loks. If there was a loose omentum, the omentum was pulled up and fixed with a Hem-o-lok (Figure 2).

**Figure 3.**
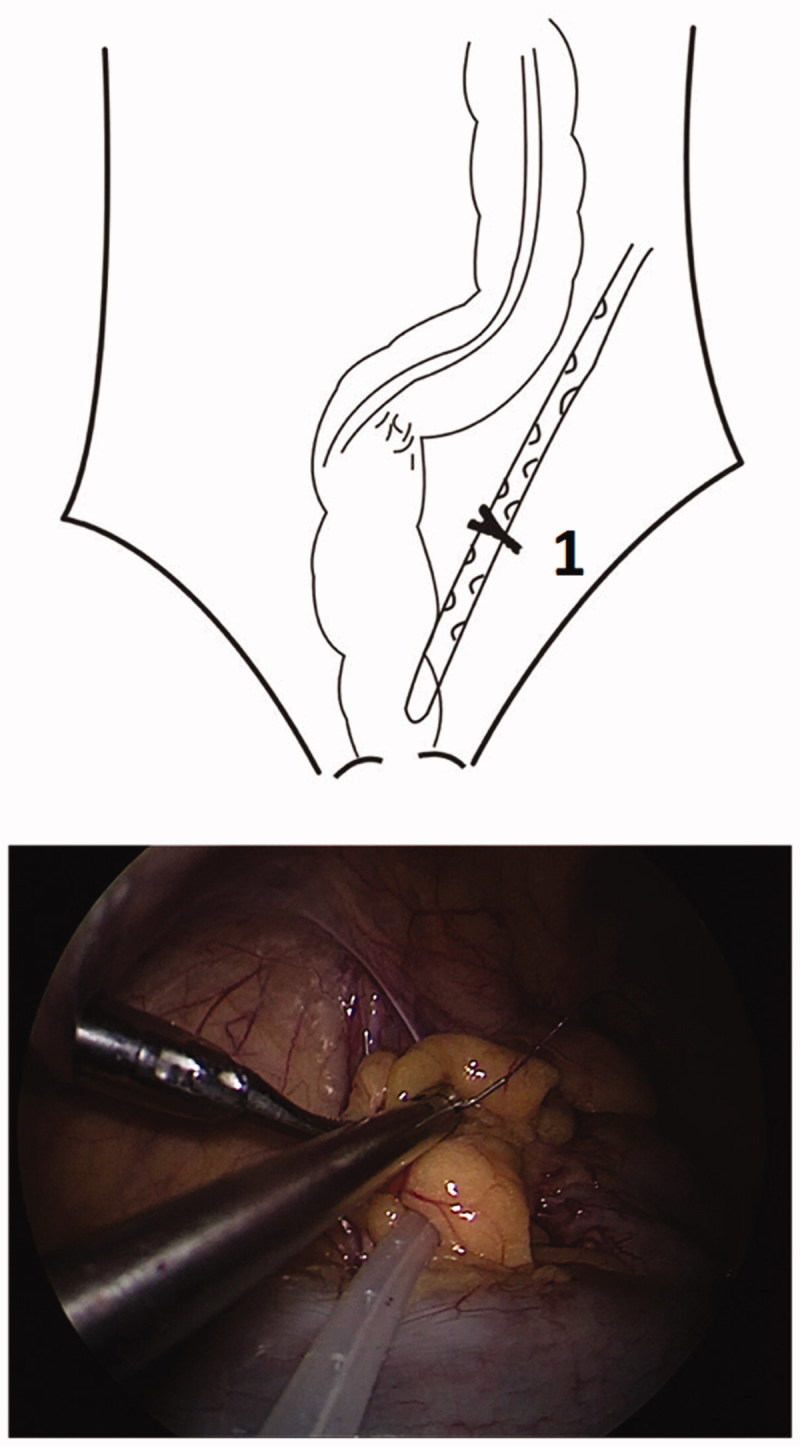
Putting and fixing the PD catheter through the left side of sigmoid colon below the suture between sigmoid mesentery and the lateral peritoneum^1^. (The top is a diagram and the bottom is laparoscopic view).

With the patient in a low-head and high-hip position, the small intestine was expelled to expose the pelvic cavity. A 3–0 suture was used to fix the sigmoid mesentery and the lateral peritoneum. With point A trocar serving as the observation port, PD catheter was inserted through point B trocar into the Douglas fossa through the left side of the sigmoid colon below the suture between sigmoid mesentery and the lateral peritoneum (Figure 3).

**Figure 4.**
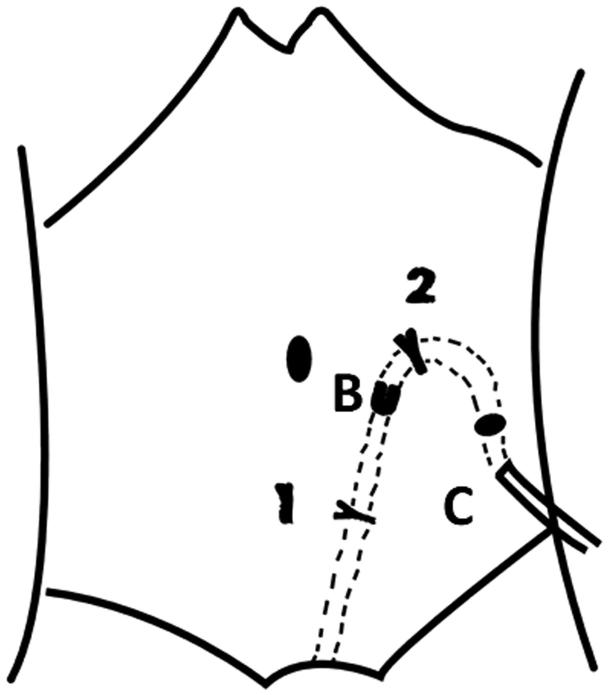
Building a subcutaneous catheter tunnel and suturing the anterior sheath of the rectus abdomini above the point B^2^.

Under laparoscopy, the inner cuff was placed in the posterior sheath of the rectus abdominis, then all trocars were withdrawn. A subcutaneous catheter tunnel was separated between point B and point C. The catheter was made into a natural arc. The catheter run through the tunnel to point C, the outer cuff was in subcutaneous tunnel, about 2–3 cm away from point C. Finally, the anterior sheath of rectus abdominis was sutured up. All incisions were closed (Figure 4).

### Postoperative care and evaluation

PD fluid (0.5 L) was irrigated into the abdominal cavity through PD catheter during the first three days after surgery. PD was performed on the fourth postoperative day. PD regimen was transformed from ambulatory peritoneal dialysis to continuous ambulatory peritoneal dialysis. The catheter’s location was detected with plain abdominal radiography. All patients’ complications were followed up, including PD-related infections, flow failures and leakage.

### Definitions

Exit-site infection was defined as the presence of erythema and purulent discharge at the catheter’s exit site. Catheter leakage was defined as the leakage of dialysis fluid from the exit site. Outflow and inflow failures were defined as no free outward and inward flow of the dialysis fluid in the catheter.

### Statistical analysis

Statistical analysis was performed using SPSS version 20.0 for Windows (IBM, USA). Categorical variables were presented as counts and percentages. Continuous variables were presented as mean ± standard deviation.

## Results

### Demographic and clinical characteristics of ESRD patients

Demographic and clinical characteristics of ESRD patients were shown in Table 1. Ten patients were included: 6 males and 4 females, aged 30–76 years, mean age of 50.0 ± 15.9 years, average body mass of 22.5 ± 2.7, and eGFR of 6.0 ± 2.0 mL/(min × 1.73 m^2^). As to the primary etiology of their renal failure, hypertension was found in three patients, chronic glomerulonephritis in five, diabetic nephropathy in one and obstructive nephropathy in one. Two patients had undergone abdominal surgery before.

**Table 1.** The demographic and clinical characteristics of ESRD patients.

Characteristic	Patients with ESRD
*N*	10
Men, *n*（%）	6（60）
Age, yr	50.0 ± 15.9
Body mass	22.5 ± 2.7
eGFR (MDRD) ml/(min × 1.73 m^2^)	6.0 ± 2.0
Previous abdominal surgery, *n*（%）	2（20）
Causes of ESRD, *n*（%）	
Chronicglomerular nephritis	5(50)
Diabetic nephropathy	1(1)
Hypertension renal disease	3(30)
Obstructive nephropathy	1(1)

### Operative process and postoperative complications

As shown in Table 2, operative time was 50.6 ± 15.4 min (range of 30–80 min). No bleeding appeared in all ten patients. In two patients with incision leakage, the conservative regimen and one-week hemodialysis was given, PD was not performed until the wound complete healing. After a period of follow-up, PD was performed routinely without leakage recurrent again. Catheter flow obstruction, migration, exit-site infection were not detected in our study. All patients were followed up for a mean of 6.0 ± 4.7 months, ranging from 1 to 15 months.

**Table 2.** Operative time and postoperative complications.

Operative and postoperative situations	Patients with ESRD
Operative time(minute)	50.6 ± 15.4
Postoperative follow-up（month）	6.0 ± 4.7
Postoperative complications,*n*（%）	
Bleeding	0 (0)
Incision leakage	2 (20)
Catheter flow obstruction	0 (0)
Exit-siteinfection	0 (0)
Catheter flow obstruction	0 (0)

## Discussion

Owing to its low cost and high operability, PD has become a common treatment for patients with ESRD. The way of PD catheter placement influences catheter function, incidence of catheter-related complications and catheter survival. Although PD catheter insertion technique has been continuously improved since its introduction more than 40 years ago, no consensus has been reached. Up to now, open catheter insertion and laparoscopic catheter insertion have been reported. Open catheter insertion, more popular, refers to the blind placement of the catheter tip into the pelvis through an open incision. This technique cannot allow surgeons to perform adhesiolysis, and also comes with a high risk of catheter dysfunction, including catheter tip migration, pericatheter leakage, catheter flow dysfunction, etc. The incidence of catheter dysfunction was up to 36% in open insertions [8,9,12,13]. One meta-analysis compared open insertion with the laparoscopic insertion, finding the later significantly reduced the incidence of catheter complications [14]. One cause of catheter flow dysfunction is the obstruction of catheter’s side holes by the omentum. Besides, omental entrapment can cause catheter obstruction or dislocation that leads to suboptimal drainage [9,15].

Laparoscopic insertion ensures the accurate placement of PD catheter into the Douglas fossa. When adhesions exist in the abdominal cavity, the adhesiolysis can be used to prevent mechanical catheter complications [16]. Laparoscopic technique can also assess the condition of the omentum, including its volume and length. Proactive techniques such as omentectomy and omentopexy can be used to prevent omental entrapment and increase catheter survival [9,17].

McIntosh et al. first used an ‘omental hitch’ technique to prevent the entrapment of PD catheter in 1985 [18]. The incidence of catheter obstruction was only 17% in their study. They sutured the free edge of the omentum to the abdominal wall in the epigastric region through an open incision. O˘günc described a new omentopexy in which two auto suture tackers were used to fix the lateral inferior edges of the omentum onto the parietal peritoneum of the lateral abdominal wall during the catheter implantation [12]. In 2013, Gilles Dupre reported a laparoscopic-assisted PD catheter insertion: in dogs, the laparoscopic-assisted partial omentectomy and omentopexy were performed after placing one sub-umbilical laparoscope portal and one instrument portal in the left cranial abdominal quadrant [11]. In the latest literature, a surgical technique was introduced, in which the omentopexy, PD catheter insertion and the rectus sheath tunneling were done using a single PD port which can reduce port-site complications and dialysis fluid leakage [19].

Having weighed the advantages of the above-mentioned techniques, we designed this laparoscopic technique using Hem-o-loks for omentopexy to fix PD catheter through the left side of sigmoid colon below the suture between sigmoid mesentery and the lateral peritoneum. This technique was effective for prevention of mechanical complications of the PD catheter.

In our study, there were two patients with leakage. To explain this problem, some literature has been looked up, in which, showed rectus sheath tunneling can further reduce leakage [19,20], and this method will be added in our future study. Laparoscopic placement of PD catheter is safer and easier than traditional techniques. As a recommendation, future studies may compare the technique described in this study with conventional open techniques.
